# The Biotechnological Potential of Plant Growth-Promoting Rhizobacteria Isolated from Maize (*Zea mays* L.) Cultivations in the San Martin Region, Peru

**DOI:** 10.3390/plants13152075

**Published:** 2024-07-26

**Authors:** Winston Franz Ríos-Ruiz, Rosslinn Esmith Tarrillo-Chujutalli, Jose Carlos Rojas-García, Cicerón Tuanama-Reátegui, Danny Fran Pompa-Vásquez, Carlos Alberto Zumaeta-Arévalo

**Affiliations:** 1 Laboratorio de Microbiología Agrícola “Raúl Ríos Reátegui”, Departamento Académico Agrosilvopastoril, Facultad de Ciencias Agrarias, Universidad Nacional de San Martín, Tarapoto 22202, Peru; rosslinn12@gmail.com (R.E.T.-C.); jogarcia@unsm.edu.pe (J.C.R.-G.); df.pompava@unsm.edu.pe (D.F.P.-V.); ca.zumaetaar@unsm.edu.pe (C.A.Z.-A.); 2 Departamento Académico de Ingeniería Agroindustrial, Facultad de Ingeniería Agroindustrial, Universidad Nacional de San Martín, Tarapoto 22202, Peru; ctuanamar@unsm.edu.pe

**Keywords:** plant growth-promoting rhizobacteria, PGPR, biotechnology, biofertilizer, *Peribacillus* sp., *Sporosarcina* sp.

## Abstract

Maize (*Zea mays* L.) is an essential commodity for global food security and the agricultural economy, particularly in regions such as San Martin, Peru. This study investigated the plant growth-promoting characteristics of native rhizobacteria isolated from maize crops in the San Martin region of Peru with the aim of identifying microorganisms with biotechnological potential. Soil and root samples were collected from maize plants in four productive zones in the region: Lamas, El Dorado, Picota, and Bellavista. The potential of twelve bacterial isolates was evaluated through traits, such as biological nitrogen fixation, indole acetic acid (IAA) production, phosphate solubilization, and siderophore production, and a completely randomized design was used for these assays. A completely randomized block design was employed to assess the effects of bacterial strains and nitrogen doses on maize seedlings. The B3, B5, and NSM3 strains, as well as maize seeds of the yellow hard ‘Advanta 9139’ variety, were used in this experiment. Two of these isolates, B5 and NSM3, exhibited outstanding characteristics as plant growth promoters; these strains were capable of nitrogen fixation, IAA production (35.65 and 26.94 µg mL^−1^, respectively), phosphate solubilization (233.91 and 193.31 µg mL^−1^, respectively), and siderophore production (34.05 and 89.19%, respectively). Furthermore, molecular sequencing identified the NSM3 isolate as belonging to *Sporosarcina* sp. NSM3 OP861656, while the B5 isolate was identified as *Peribacillus* sp. B5 OP861655. These strains show promising potential for future use as biofertilizers, which could promote more sustainable agricultural practices in the region.

## 1. Introduction

Maize (*Zea mays* L.) is a globally significant food product, used for both human and animal consumption. In Peru, approximately 520,000 hectares are planted annually nationwide, with around 82,000 families directly dependent on this crop. Of this area, approximately 300,000 hectares are dedicated to yellow maize and 220,000 hectares to amylaceous maize [1]. In the San Martin region of Peru, there were 68,473.50 hectares planted with yellow maize in the 2023 season, with a production of 262,227.30 tons per year and yields averaging 3.83 tons per hectare, which is considered low compared to the national average of 4.84 tons per hectare [2]. This low yield may be attributed to factors, such as physical, chemical, and biological soil degradation, inadequate farming practices, the lack of crop rotation, the presence of pests and diseases, and the excessive use of agrochemicals, all of which result in various health and environmental issues [3].

An alternative to the excessive use of chemical inputs is biofertilizers, which consist of plant growth-promoting rhizobacteria (PGPR). These rhizobacteria, through their nutritional profile, represent one of the most employed strategies for selecting microorganisms with biotechnological interest to develop alternatives to synthetic chemical fertilizers. It is estimated that diazotrophic bacteria can fix up to 82% of the nitrogen required by maize crops during their phenological period [4]. Investigating PGPR in maize crops in San Martin, Peru, is relevant and timely due to their potential to increase agricultural productivity, promote environmental sustainability, and improve local food security in a region where agriculture is vital for livelihood and development [5,6,7]. The search for new strains of microorganisms capable of promoting growth is a sustainable option for maize cultivation. Native PGPR are specifically adapted to the soil and climate of their regions, allowing them to function more efficiently and provide more consistent benefits than introduced strains [8]. They acquire their adaptability and resilience through co-evolution [9], resistance to biotic and abiotic stress [10], production of bioactive compounds [11], genetic diversity [12], microbial interactions [13], and natural selection [14]. These mechanisms enable them to play a crucial role in promoting plant growth and agricultural sustainability in tropical rainforest environments.

Identifying the characteristics of PGPR, such as the biological nitrogen fixation, phosphate solubilization, siderophore production, and hormone production, all of which are evaluated in this study, provides knowledge supporting their use as inoculants in this crop. The increasing application of PGPR sustainably improves the yield of key crops like maize, and their large-scale use could reduce the need for harmful pesticides and fertilizers, contributing to environmental preservation [15].

Various studies have addressed the beneficial effects of rhizobacteria on plant growth and yield. These investigations are widely described in reviews [16,17], and specifically in maize in [7,18]. Singh et al. [19] isolated strains of *Pseudomonas*, *Rhizobium*, *Bacillus*, and *Enterobacter* from the maize rhizosphere, which showed nutrient solubilization activities, indole acetic acid production, cell wall hydrolyzing enzymes (cellulase and pectinase), and siderophore production. Oliveira et al. [20] and Moreira et al. [21] evaluated the inoculation of *Azospirillum brasiliense* in maize cultivation, determining an increase in maize yield compared to non-inoculated plants fertilized with nitrogen. Abdel Latef et al. [22] evaluated the effects of *Azospirillum lipoferum* and *Azotobacter chroococcum* on maize cultivation and observed that they could mitigate the negative effects of salt stress on maize plants. Javoreková et al. [23] isolated and characterized a total of eleven plant growth-promoting rhizobacteria from the maize rhizosphere grown in luvisols, identifying three *Bacillus* species that could be used as biofertilizers to increase maize production. Similarly, Sukweenadhi et al. [24] isolated plant growth-promoting bacteria from maize, identifying them as *Bordetella muralis*, *Cellulosimicrobium cellulans*, and *Serratia nematodiphila*. Recently, the efficacy of *Bacillus subtilis* strains capable of forming biofilms as plant growth promoters in *Zea mays* L., as well as their ability to produce secondary metabolites with biocidal effects, has been evaluated [25]. Furthermore, Ercole et al. [26] investigated the ability of these strains to tolerate high salt concentrations in maize cultivation. Galindo et al. [7] evaluated the impact of nitrogen fertilizer sustainability on maize crop yield through the co-inoculation of *Azospirillum brasilense* and *Bacillus subtilis*, which played a crucial role in nitrogen recovery.

Research on rhizobacteria that have adapted to various edaphoclimatic environments and their beneficial effects on food crops has sparked growing interest due to their significant biotechnological potential [27,28]. The production of inoculants with native PGPR strains can enhance their effectiveness and adaptability to various conditions, reducing the reliance on chemical fertilizers and promoting agricultural sustainability and soil health. The purpose of this study was to investigate the native rhizobacteria present in the maize rhizosphere in the San Martin region, with a view to their possible future application as biofertilizers.

## 2. Results

### 2.1. Isolation of Rhizobacteria from Maize Plants

Twelve rhizobacteria were isolated from the rhizosphere of maize plants from the provinces of Lamas, El Dorado, Picota, and Bellavista, using two nitrogen-free culture media, JMV and Burk. The proportion of Gram-positive to Gram-negative rhizobacteria was 58.3% to 41.7%, with the majority being bacilli (83.3%) and a smaller proportion being cocci (16.7%) (Table 1).

All isolated rhizobacteria were able to grow in nitrogen-free JMV and Burk media, demonstrating their capability for biological nitrogen fixation. This was evidenced by the formation of a film on the surface of semi-solid media after a 45-day incubation period.

### 2.2. Promoters of Plant Growth Parameters Produced by Rhizobacteria

#### 2.2.1. Production of Indole Acetic Acid (IAA)

All the evaluated strains produced IAA within a highly variable range, from 1.15 to 44.54 µg IAA mL^−1^ of TSB medium (Table 2). Notably, strains B3 and P4, originating from the Picota province, sectors of Barranquita and Ponaza, respectively, produced the highest amounts of IAA, with 44.54 and 41.59 µg IAA mL^−1^ of TSB, respectively. In contrast, strains L3-1 and L6, from the Lamas province, Alto Pucalpillo sector, produced lower amounts of IAA, with levels of 1.15 and 4.57 µg IAA mL^−1^, respectively.

#### 2.2.2. Solubilization of Aluminum Phosphate (AlPO_4_)

All the isolated strains solubilized AlPO_4_ (Table 2). The strains B5 and SJ1 from the provinces of Picota, Barranquita sector, and Bellavista, San Jose sector, showed the highest solubilization levels, with indices of 233.91 and 224.08 μg mL^−1^, respectively. In comparison, strains L3-1 and L2 from the province of Lamas, Alto Pucalpillo sector, exhibited significantly lower solubilization capacity, with indices of 146.30 and 150.57 μg mL^−1^, respectively.

#### 2.2.3. Production of Siderophores

All the strains produced siderophores (Table 2). Strains NSM3 and P4 from the provinces of El Dorado, Nuevo San Martin sector, and Picota, Ponaza sector, showed the highest production levels with indices of 89.191 and 57.12%, respectively. In comparison, strains L3-1 and L6 from the province of Lamas, Alto Pucalpillo sector, exhibited significantly lower production, with indices of 2.70 and 3.06%, respectively.

### 2.3. Seed Germination Assay

**Figure 1:**
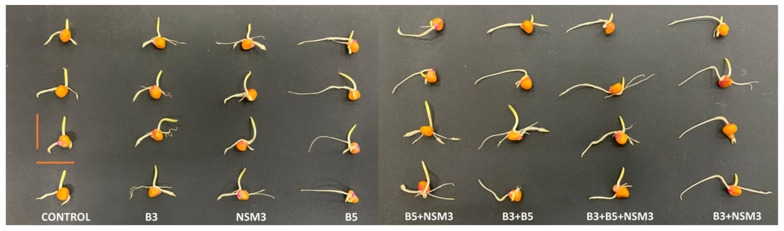
Effect of the inoculation with strains B3, B5, NSM3, and their combinations (B3 + B5; B3 + NSM3; B5 + NSM3; B3 + B5 + NSM3) on the germination of *Zea mays* L. var. ‘Advanta 9139’, compared to the uninoculated control, at 72 h after inoculant application. The red bars indicate a scale of 4 cm in length.

The results obtained at 72 h after inoculant application to the seeds showed that the treatments containing inoculum B5 and the combination B3 + NSM3 produced greater root length compared to other treatments and the uninoculated control (Figure 1). Regarding the germination percentage, all the treatments exceeded 95% seed germination.

### 2.4. Gnotobiotic Experiment

**Figure 2:**
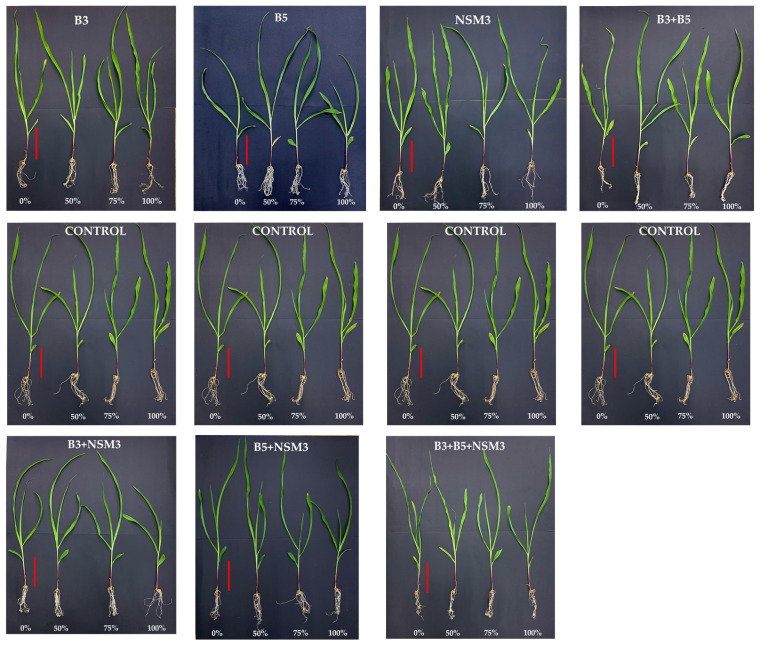
Effect of the individual inoculation with strains B3, B5, and NSM3, as well as their combinations (B3 + B5, B3 + NSM3, B5 + NSM3, and B3 + B5 + NSM3), under different nitrogen doses (0%, 50%, 75%, and 100%) on maize seedlings grown under gnotobiotic conditions 15 days post-inoculation. The control treatment was conducted without any strains. Each image shows the phenotype of one representative seedling out of six replicates per treatment. The vertical red bar in each image represents a scale length of 10 cm.

From a general perspective, the morphological evaluations conducted at the end of the experiment (15 days after inoculation) revealed that the seedlings from the inoculated treatments showed higher results in the various parameters evaluated compared to the uninoculated control. It was determined that all the treatments increased the studied parameters compared to the uninoculated control. The root length was influenced by the shape and size of the container (50 mL Falcon tubes), resulting in a homogeneous shape in most treatments (Figure 2).

**Figure 3:**
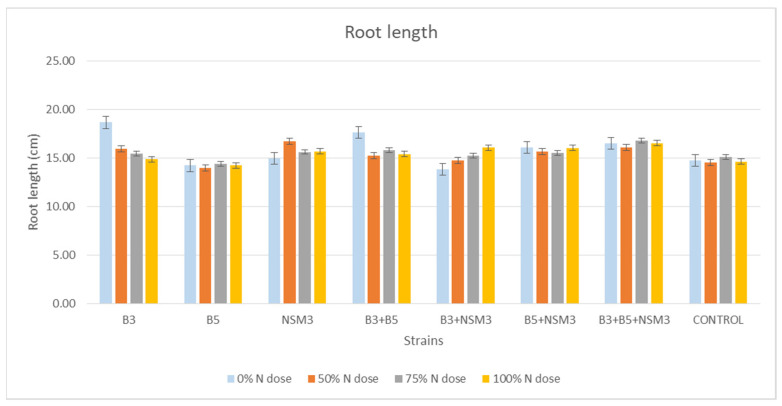
Effect of inoculation with strains B3, B5, and NSM3, as well as their combinations (B3 + B5, B3 + NSM3, B5 + NSM3, and B3 + B5 + NSM3), under different nitrogen doses on the root length of maize seedlings (*Zea mays* L.) grown under gnotobiotic conditions, evaluated 15 days post-inoculation.

At 15 days after inoculation, significant variations in the maize seedling root lengths were observed across the different treatments of strains and their combinations, as well as nitrogen doses. At a 0% nitrogen dose, strain B3 showed the longest root length (18.65 cm), followed by the combination B3 + B5 (17.65 cm). At a 50% dose, strain NSM3 exhibited the longest length (16.70 cm). At 75 and 100% nitrogen doses, the combination of B3 + B5 + NSM3 showed the longest length (16.78 and 16.53 cm, respectively) (Figure 3 and Table S1).

**Figure 4:**
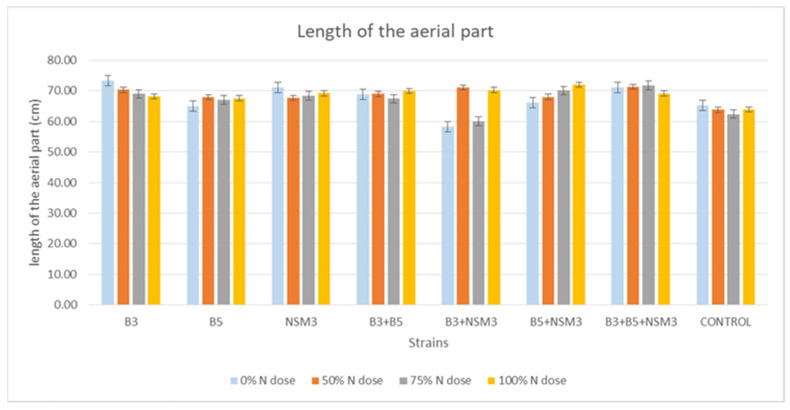
Effect of inoculation with strains B3, B5, and NSM3, as well as their combinations (B3 + B5, B3 + NSM3, B5 + NSM3, and B3 + B5 + NSM3), under different nitrogen doses on the length of the aerial part of maize seedlings (*Zea mays* L.) grown under gnotobiotic conditions, evaluated 15 days post-inoculation.

Regarding the length of the aerial part, at the 0% nitrogen dose, strain B3 showed the greatest development (73.38 cm) compared to other treatments and the control. At 50 and 75% nitrogen doses, the combination B3 + B5 + NSM3 exhibited the greatest development (71.30 and 71.82 cm, respectively) compared to other treatments and the control. At a 100% nitrogen dose, the combination of strains B5 + NSM3 stood out (72.05 cm) compared to other treatments and the control (Figure 4 and Table S1).

**Figure 5:**
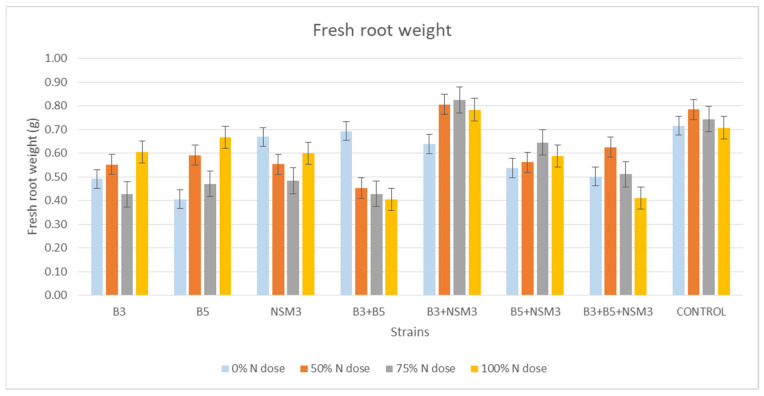
Effect of inoculation with strains B3, B5, and NSM3, as well as their combinations (B3 + B5, B3 + NSM3, B5 + NSM3, and B3 + B5 + NSM3), under different nitrogen doses on the fresh root weight of maize seedlings (*Zea mays* L.) grown under gnotobiotic conditions, evaluated 15 days post-inoculation.

The fresh root weight showed significant differences among treatments. The combination B3 + NSM3 exhibited a high weight at all nitrogen doses, particularly at 50, 75, and 100% nitrogen doses with values of 0.805, 0.824, and 0.783 g, respectively (Figure 5 and Table S1).

**Figure 6:**
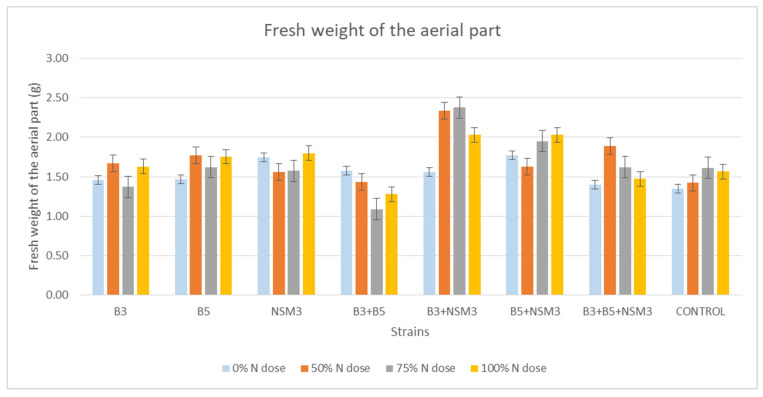
Effect of inoculation with strains B3, B5, and NSM3, as well as their combinations (B3 + B5, B3 + NSM3, B5 + NSM3, and B3 + B5 + NSM3), under different nitrogen doses on the fresh weight of the aerial part of maize seedlings (*Zea mays* L.) grown under gnotobiotic conditions, evaluated 15 days post-inoculation.

Regarding the fresh weight of the aerial part, the combination B3 + NSM3 showed the highest fresh weight at the 50 and 75% nitrogen doses, with values of 2.339 and 2.376 g, respectively, and the combination B5 + NSM3 at the 100% dose, with a value of 2.030 g. The control exhibited the lowest fresh weights for all nitrogen doses, except at the 0% dose, where it was comparable to the individual strains (Figure 6 and Table S1).

**Figure 7:**
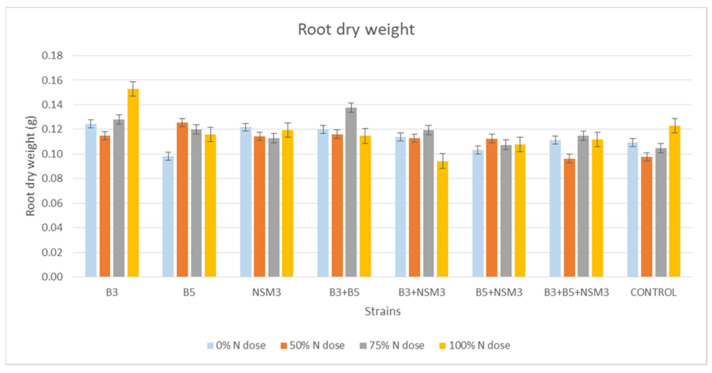
Effect of inoculation with strains B3, B5, and NSM3, as well as their combinations (B3 + B5, B3 + NSM3, B5 + NSM3, and B3 + B5 + NSM3), under different nitrogen doses on the root dry weight of maize seedlings (*Zea mays* L.) grown under gnotobiotic conditions, evaluated 15 days post-inoculation.

**Figure 8:**
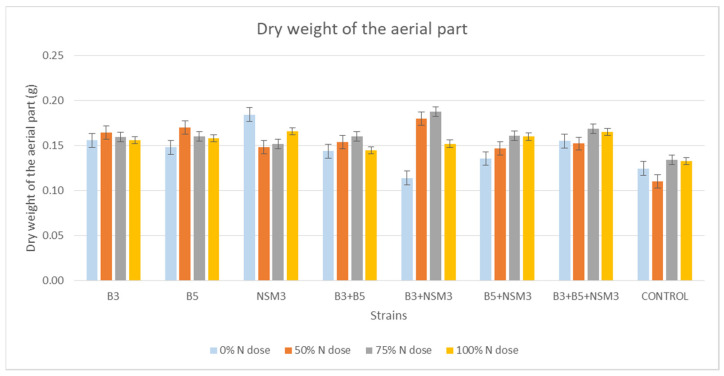
Effect of inoculation with strains B3, B5, and NSM3, as well as their combinations (B3 + B5, B3 + NSM3, B5 + NSM3, and B3 + B5 + NSM3), under different nitrogen doses on the dry weight of the aerial part of maize seedlings (*Zea mays* L.) grown under gnotobiotic conditions, evaluated 15 days post-inoculation.

Regarding the dry weight of the root, no significant differences were observed between treatments with bacterial strains and different nitrogen doses compared to the control (Figure 7). However, in terms of the dry weight of the aerial part, all treatments containing bacterial strains with different nitrogen doses were higher than the uninoculated control (Figure 8).

### 2.5. Molecular Identification of Isolates

**Figure 9:**
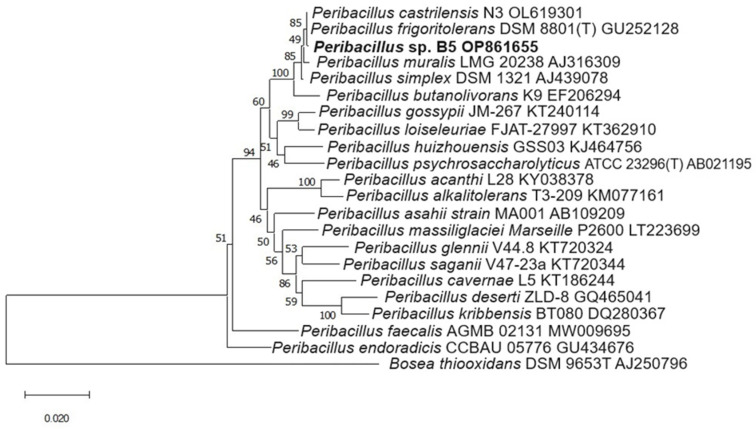
Neighbour-joining phylogeny based on 16S rRNA gene sequences (1572 positions) showing relationships among *Peribacillus* species. Branch support values are indicated as bootstrap percentages calculated from 1000 subsets (only values above 50% are shown). Scale bar, 1 substitution per 100 nucleotide positions. The 16S rRNA gene sequence of *Bosea thiooxidans* DSM 9653T was used as an outgroup. The phylogenetic tree includes the identified species *Peribacillus* sp. B5 OP861655, highlighting its relationship with other *Peribacillus* species.

**Figure 10:**
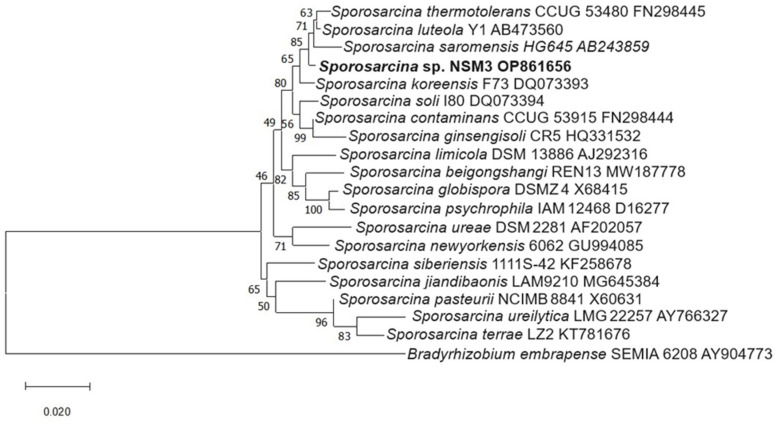
Neighbour-joining phylogeny based on 16S rRNA gene sequences (2169 positions) showing relationships among *Sporosarcina* species. Branch support values are indicated as bootstrap percentages calculated from 1000 subsets (only values above 50% are shown). Scale bar, 2 substitutions per 100 nucleotide positions. The 16S rRNA gene sequence of *Bradyrhizobium embrapense* SEMIA 6208 was used as an outgroup. The phylogenetic tree includes the identified species *Sporosarcina* sp. NSM3 OP861656, highlighting its relationship with other *Sporosarcina* species.

Figure 9 and Figure 10 show the phylogenetic trees of the rhizobacteria *Peribacillus* sp. B5 OP861655 and *Sporosarcina* sp. NSM3 OP861656, obtained using the 16S rRNA gene sequences of each.

The BLAST analysis revealed that the isolates belong to the genera *Peribacillus* and *Sporosarcina* (Table 3). Isolate B5 from Picota Province, Barranquita sector, showed a close relationship with *Peribacillus frigoritolerans* DSM 8801T, with 99.93% identity. Similarly, isolate NSM3 from El Dorado Province, Nuevo San Martin sector, exhibited high similarity with *Sporosarcina luteola* Y1 AB473560, with 99.94% identity. The molecular datasets generated were deposited in the GenBank of the National Center for Biotechnology Information (NCBI) (http://www.ncbi.nlm.nih.gov/genbank/, accessed on 20 November 2022) under their corresponding accession numbers (Table 3).

## 3. Discussion

### 3.1. Isolation of Rhizobacteria from Maize Plants

The substantial number of growth-promoting rhizobacteria isolated in this study was consistent with that in previous research. For example, Martínez and Quispe [29] isolated bacterial strains with nitrogen-fixing properties from different maize plant organs after 1, 2, and 3 months of development. Similarly, Rodríguez-Hernández et al. [30] evaluated five Bacillus spp. strains from maize root systems, and all exhibited nitrogen-fixing potential. Zhu et al. [31] isolated Bacillus megaterium OQ560352 from the maize rhizosphere, which promoted growth in saline soils by altering rhizosphere microbial communities and enhancing organic phosphorus utilization. The nitrogen-fixing rhizobacteria isolated from maize rhizospheres in the San Martin region are important due to their ability to convert atmospheric nitrogen into forms usable by plants. This process sustainably enhances crop growth and productivity, promotes environmentally friendly agricultural practices, and supports regional food security.

### 3.2. Plant Growth-Promoting Parameters Produced by Rhizobacteria

#### 3.2.1. Production of IAA

The production of phytohormones such as IAA is a key mechanism in plant growth-promoting rhizobacteria (PGPR), which are essential for plant development [32]. Parra-Cota et al. [33] reported auxin production by bacteria from maize crops ranging from 2.00 to 12.33 μg mL^−1^. Bolívar-Anillo et al. [34] found that strains 5Cs and 5Cm had high IAA production levels of 3.7 and 2.6 μg mL^−1^, respectively. Navid et al. [35] studied *Bacillus simplex* and its role in maize growth by analyzing auxin biosynthesis. In this study, all evaluated strains produced IAA, with strains B3 and P4 yielding the greatest amounts (44.54 and 41.59 µg mL^−1^, respectively). Agunbiade et al. [36] also found IAA production in all eleven rhizobacterial strains isolated from maize under drought conditions. The presence of IAA-producing bacteria in the maize rhizosphere of the San Martin region offers a significant opportunity to sustainably improve local agriculture, benefiting both farmers and the environment.

#### 3.2.2. Solubilization of AlPO_4_

Growth-promoting rhizobacteria enhance plant nutrition by solubilizing insoluble phosphates in soil, thus increasing phosphorus uptake [37,38]. Pérez-Pérez et al. [39] reported similar findings, isolating fifteen bacterial strains capable of solubilizing various phosphate sources. In contrast, Azizah et al. [40] found lower phosphate solubilization levels, with isolates AP1.3 and BP1.3 solubilizing 12.07 and 11.09 μg mL^−1^, respectively, compared with 146.30 to 233.91 μg mL^−1^ in this study. Phosphate solubilization ability varies by bacterial type, environment, and organic acid production [40]. Additionally, Sangoquiza-Caiza et al. [41] characterized diverse nitrogen-fixing and phosphate-solubilizing bacteria from maize root systems in Ecuador, while Agunbiade et al. [36] found that eleven IAA-producing bacterial strains from the maize rhizosphere also solubilized phosphate.

#### 3.2.3. Siderophore Production

Siderophore production is essential for bacteria to sequester iron and supply it to plants through specific transport systems and cell membrane receptors [42]. This study’s results align with those of Parra-Cota et al. [43], who noted high siderophore production potential in maize-derived bacterial strains. Similarly, Bolívar-Anillo et al. [34] found that all *Bacillus subtilis* strains from the maize rhizosphere produced siderophores and could grow in nitrogen-free media. Agunbiade et al. [36] also observed that eleven maize rhizosphere strains that were positive for IAA and phosphate solubilization produced siderophores. In this study, the NSM3 and P4 strains were notable for their siderophore production, with concentrations of 89.19% and 57.12%, respectively (Table 2). This capacity for siderophore production in NSM3 and P4 offers a promising approach to sustainable agriculture by improving iron availability and enhancing plant growth and health.

### 3.3. Germination Assay

At 72 h post-inoculation, strain B5 and the B3 + NSM3 combination exhibited significantly greater root lengths than those of the other treatments and the control (Figure 1). This indicated that these treatments enhanced root growth in *Zea mays* L. var. ‘Advanta 9139’. The enhanced root growth was attributed to the high IAA production of the bacterial strains, with strain B5 producing 35.65 μg mL^−1^ and the B3 + NSM3 combination producing a total of 71.48 μg mL^−1^ (44.54 μg mL^−1^ from B3 and 26.94 μg mL^−1^ from NSM3), as shown in Table 2. IAA is an auxin that is critical for root development; these strains’ IAA levels effectively stimulate root growth [44]. Moreover, the results highlight the benefit of bacterial strain combinations in promoting plant growth, with the B3 + NSM3 combination proving especially effective due to its synergistic effect on IAA production and root growth [45].

### 3.4. Gnotobiotic Experiment

Inoculation with PGPR strains significantly improved the root length, aerial part length, fresh root weight, fresh weight of the aerial part, and dry weight of the aerial part compared with the uninoculated control, although it did not affect the root dry weight. Under 0% nitrogen, strain B3 had the longest roots, while strain NSM3 excelled at 50% nitrogen. The combination B3 + B5 + NSM3 was superior at 75% and 100% nitrogen. For the length of the aerial part, strain B3 performed best under 0% nitrogen, B5 + NSM3 was optimal at 100% nitrogen, and B3 + B5 + NSM3 was effective at 50% and 75% nitrogen. These results align with those of other studies showing that PGPR, such as *Pseudomonas putida* and *Bacillus subtilis*, promote better root and shoot growth [46]. The combination B3 + NSM3 showed the highest fresh root and aerial weights across the different nitrogen levels, indicating potential synergy and suggesting that bacterial consortia can optimize crop growth by integrating mechanisms such as nitrogen fixation and IAA production [6]. Despite there being no significant differences in root dry weight, the bacterial treatments improved the aerial biomass, supporting previous findings of enhanced biomass with *Herbaspirillum seropedicae* under high nitrogen [47].

These results highlight the potential of PGPR to improve plant performance and reduce nitrogen fertilizer use, with B3, B5, and NSM3 showing notable benefits. This study is pioneering in its identification of native PGPR strains in San Martin’s tropical environment. Further research is needed to explore these strains’ mechanisms and effectiveness in various soils and conditions [48].

### 3.5. Molecular Identification of Isolates

Among the twelve isolated rhizobacterial strains, two were molecularly identified: strain B5 as *Peribacillus* sp. B5 OP861655 and strain NSM3 as *Sporosarcina* sp. NSM3 OP861656. *Peribacillus*, a Gram-positive bacillus, includes species such as *Peribacillus simplex,* which promotes plant growth and acts as a biocontrol agent [49,50]. *Sporosarcina* bacteria, which are also Gram-positive and bacillus-shaped [51], are known for their urease production and role in the nitrogen cycle [52]. In our study, *Sporosarcina* sp. NSM3 enhanced maize seedlings’ dry weight under nitrogen-free conditions and, when combined with strains B3 and B5, increased the dry weight at 75% nitrogen, suggesting its role in biological nitrogen fixation under low-nitrogen conditions.

## 4. Materials and Methods

### 4.1. Isolation of Rhizobacteria from Maize Plants

**Figure 11:**
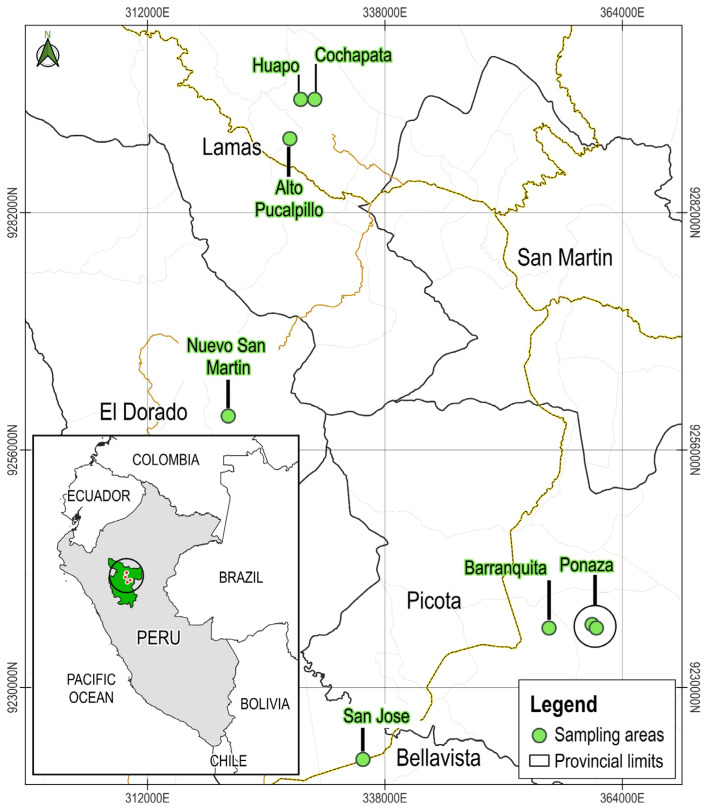
Map showing the provinces of Lamas, El Dorado, Picota, and Bellavista within the San Martín region of Peru. The marked locations indicate the specific sites visited for the collection of rhizospheric soil and maize root samples. The circle on the inset map of Peru highlights the San Martín region, pinpointing the collection zones.

Samples of soil and rhizospheric roots from maize plants were collected following the methodology proposed by Burt [53] in four production zones of the San Martin region, Peru: Lamas, El Dorado, Picota, and Bellavista (Figure 11). Table 4 shows the specific locations of the collection zones within each production area.

In each zone, six vigorous and healthy plants of hard yellow maize were sampled before the flowering stage. For the isolation of rhizobacteria, samples were analyzed within 24 h after collection following the methodology described by Ríos-Ruiz et al. [5]. First, the roots were washed in distilled water and cut into approximately 0.5 cm pieces using scissors; then, they were dried with filter paper. Next, 0.2 g of roots was placed in 1.5 mL microtubes (minimum of three replicates). Subsequently, 1 mL of sterile saline solution (0.9% NaCl) was added to each microtube, vortexed for 20 s to release the rhizospheric soil, and then centrifuged at 2000 rpm in a centrifuge (Mikro200 Hettich, Tuttlingen, Germany), collecting the supernatant in another 1.5 mL microtube. Finally, 200 µL of the supernatant was placed in 50 mL Falcon tubes filled to 2/3 of their volume with semi-solid culture media, free of combined nitrogen, JMV, and Burk. The sealed Falcon tubes were then incubated at 30 °C until a bacterial growth film appeared. The growth became visible after 45 days of incubation. Subsequently, the upper layer of the culture medium was removed using a sterile spatula. The bacterial layer grown on the three-quarters of the tube was then transferred to 50 mL Falcon tubes, adding sterile saline solution to each Falcon tube and homogenizing the sample using a vortex mixer for 1 min. Serial dilutions up to the tenth dilution were prepared in saline solution, with 100 µL of each dilution inoculated onto Petri dishes containing solid culture media, either JMV or Burk. At least three replicates were performed per dilution and culture medium. Finally, the plates were incubated at 30 °C until colony forming units (CFUs) appeared.

The CFUs were selected based on their morphological characteristics through observation under a stereomicroscope (Carl-Zeiss-Promenade 10-Stemi 508, Jena, Germany). Each selected CFU was streaked onto the same medium from which it was isolated to verify the purity. Following isolation, the routine medium for bacterial culture was Tryptone Soy Agar (TSA) (pancreatic digest of casein, 15 g; papaic digest of soybean, 5 g; sodium chloride, 5 g; Agar-Agar, 15 g; distilled water, 1000 mL; pH 7.0). Phenotypic characterization included Gram staining to evaluate the individual shape and grouping. Colony characteristics, such as the shape, size, margin, color, and appearance, were also assessed.

### 4.2. Evaluation of Plant Growth-Promoting Parameters Produced by Rhizobacteria

#### 4.2.1. Determination of IAA Production

For the production of IAA, the strains were first cultivated in 2 mL of TSB liquid medium (pancreatic digest of casein, 15 g; papaic digest of soybean, 5 g; sodium chloride, 5 g; distilled water, 1000 mL; pH 7.0) and incubated at 30 °C overnight until adequate turbidity was reached. Subsequently, a portion was inoculated into tubes containing 5 mL of TSB medium, some supplemented with 100 μg mL^−1^ of tryptophan (HI-media^®^, Maharashtra, India). The tubes were then incubated at 30 °C for 24 h at 180 rpm. Afterward, 1 mL of the culture was centrifuged (Mikro200 Hettich, Tuttlingen, Germany) at 13,000 rpm for 3 min. The resulting pellet was used for protein quantification using the Bradford method, while the supernatant was used to measure the IAA production, following the methodology proposed by Gravel et al. [54]. To quantify the IAA production, 0.5 mL of the supernatant was mixed with 0.5 mL of Salkowski’s reagent by inversion. Three to four replicas of this mixture were prepared, transferred to 1 mL spectrophotometry cuvettes, and allowed to rest in darkness at room temperature for 20 min. The absorbance was measured at 535 nm using a spectrophotometer (Thermofisher Spectronic 200, Suwa, Japan), with a control consisting of 0.5 mL of culture medium plus 0.5 mL of Salkowski’s reagent. The results were expressed in μg mL^−1^.

#### 4.2.2. Determination of AlPO_4_ Solubilization

To evaluate the AlPO_4_ solubilization capacity of the strains under study, the strains were initially reactivated in TSB medium and incubated at 30 °C for 24 h. The cell suspensions were adjusted to reach an OD_600nm_ of 0.5 to form the inoculum. *Rhizobium tropici* CIAT 899 was used as a positive control [55]. AlPO_4_ solubilization was subsequently assessed following the protocol described by Ríos-Ruiz et al. [38]. Briefly, GELP medium with 0.89 g of AlPO_4_ was used to evaluate the AlPO_4_ solubilization. The solution without strains served as a blank treatment. Next, 0.5 mL of the inoculum was inoculated into 50 mL of GELP medium per strain, with three replicates, and incubated at 28 °C for 5 days with shaking at 130 rpm. After incubation, the samples were centrifuged at 13,000 rpm for 5 min, and the supernatants were stored for AlPO_4_ solubilization analysis. AlPO_4_ solubilization was determined using the phosphomolybdate method. Aliquots of 1200 µL of the culture medium were taken in triplicate, centrifuged (Mikro200 Hettich, Tuttlingen, Germany) at 13,000 rpm for 5 min, and 1000 µL of this supernatant was mixed with 120 µL of reagent solution. The mixture was incubated at room temperature for 10 min. The presence of AlPO_4_ was verified by the formation of a blue color, measured at an absorbance of 655 nm. The absorbance values were correlated with a phosphate standard curve to determine the concentration of AlPO_4_ in µg mL^−1^ in the sample.

#### 4.2.3. Measurement of Siderophore Production

The siderophore production was carried out as suggested by Sayyed et al. [56]. The rhizobacterial strains were cultured in Luria-Bertani (LB) medium (1% tryptone, 0.5% yeast extract, 1% NaCl, pH adjusted with NaOH) for 24 h at 30 °C with constant shaking at 200 rpm using a shaker (Heidolph Unimax 1010, Schwabach, Germany). Each strain was harvested, and the cells were washed three times with sterile saline solution. Subsequently, the cells were resuspended and transferred to an iron-free succinate medium (SM-Fe) (K_2_HPO_4_, 6.0 g L^−1^; KH_2_PO_4_, 3.0 g L^−1^; MgSO_4_·7H_2_O, 0.2 g L^−1^; (NH_4_)_2_SO_4_, 1.0 g L^−1^; succinic acid, 4.0 g L^−1^; and pH, 7.0). Then, the cell suspension at a concentration of 0.1% (*v*/*v*) (100 μL) was added to 10 mL of SM-Fe medium in 125 mL Erlenmeyer flasks. The flasks were incubated at 30 °C for 30 h with shaking at 170 rpm. A 1 mL aliquot from each Erlenmeyer flask containing the rhizobacterial strains was taken and centrifuged (Mikro200 Hettich, Tuttlingen, Germany) at 10,000 rpm for 15 min. The cell-free supernatant was used for siderophore quantification. Quantitative estimation of the siderophore production was performed using the CAS-Shuttle assay methodology, where 0.5 mL of culture supernatant was combined with 0.5 mL of CAS reagent. The resulting mixture was measured at 630 nm, with uninoculated broth (control) + 0.5 mL of CAS reagent as reference. To estimate the percentage of siderophore units, the following formula was applied, (Ar − As)/Ar × 100, where Ar is the absorbance of the reference, and As is the absorbance of the sample.

### 4.3. Germination Assay

To determine the effect of the rhizobacterial strains on maize seed germination, the methodology suggested by Swift [57] was employed. Inoculum of each selected strain was prepared by inoculating into tubes containing TSB medium, which were then placed on a rotary shaker at 200 rpm for 48 h at 28 °C. The cultures were adjusted to an optical density (OD) of 0.5 at 600 nm using sterile saline solution (0.89% *w/v* NaCl). Seeds were sterilized by soaking them in 70% ethanol for 1 min, followed by 4% NaOCl for 6 min; then, they were rinsed six times with sterile distilled water. For each treatment, twenty uniform seeds were placed on sterile Petri dishes with moistened sterile cotton and filter paper with sterile distilled water and incubated at room temperature for 10 h. Subsequently, bacterial suspensions corresponding to each treatment, including individual strains and combinations, were added, while TSB medium without strains was used for the control. The Petri dishes were sealed with Parafilm and incubated in the dark at 30 °C. The germination was evaluated at 24, 48, and 72 h by counting the number of germinated seeds after this period.

### 4.4. Gnotobiotic Experiment

The experiment was conducted following the protocol proposed by Etesami and Alikhani [58]. The strains B3, B5, and NSM3, along with the maize variety ‘Advanta 9139’, were used. The seeds were sterilized by soaking in 70% ethanol for 1 min, followed by 4% NaOCl for 6 min; finally, they were rinsed six times with sterile distilled water. Prior to planting, 50 mL Falcon tubes were prepared with a substrate mixture of vermiculite and river sand in a 1:1 ratio (*v*/*v*), previously washed and sterilized. To this mixture, 15 mL of Hoagland’s nutrient solution with nitrogen concentrations at 50, 75, and 100% was added using calcium nitrate tetrahydrate (Ca(NO_3_)_2_·4H_2_O). The seeds inoculated with strains B3, B5, and NSM3 were placed in prepared tubes and conditioned in a growth chamber maintained at 28 ± 2 °C, 75% relative humidity, and a 14 h light/10 h dark cycle (60 µmol m^−2^ s^−1^). The experimental design comprised completely randomized blocks, with six replications per treatment. After 15 days, the seedlings were harvested, and the parameters, such as the root length, the length of the aerial part, the fresh root weight, the fresh weight of the aerial part, the dry root weight, and the dry weight of the aerial part, were evaluated.

### 4.5. Molecular Identification of the Isolates

The amplification of the 16S rRNA gene from two strains under study was carried out using PCR reaction with the primers fD1 (5′-CCGAATTCGTCGACAACAGAGTTTGATCCTGGCTCAG-3′) and rD1 (5′-CCCGATCCAGCTTAAGGAGGTGATCCAGCC-3′) [59]. The PCR products were stained with DiamondTM and loaded onto 0.7% agarose gels prepared in TBE buffer (1×). Electrophoresis was conducted at a constant current of 60 V, and the gels were photographed under UV light. The DirectLoadTM 1 Kb DNA Ladder (Sigma Aldrich, St. Louis, MO, USA) was used as a molecular weight marker. A DNA band of approximately ~1500 base pairs was expected for the 16S rRNA gene. The amplified product was purified using the High Pure PCR Product Purification Kit (Roche) following the manufacturer’s instructions. DNA sequencing reaction mixes were sent for sequencing to Macrogen (Seoul, Republic of Korea). The sequence quality was evaluated using Chromas (v 2.6.6, http://www.technelysium.com.au/chromas14x.html, accessed on 20 September 2022), and forward and reverse sequences were assembled using Geneious Prime software (v 2023.2.1). The sequence similarity of the assembled 16S rRNA gene sequences was examined using the EZ-taxon-e server [60].

### 4.6. Statistical Analysis

A completely randomized design was used for the assays of IAA production, phosphate solubilization, and siderophore production. Additionally, a completely randomized block design was employed for the gnotobiotic assay at different levels of nitrogen fertilization over 15 days. This experiment involved strains B3, B5, and NSM3, along with maize seeds of the ‘Advanta 9139’ hard yellow variety, cultivated in Peru. Data processing and analysis utilized InfoStat/L Version 2020 statistical software, with tests for normality and homoscedasticity applied to evaluate the data.

## 5. Conclusions

In conclusion, this study of the biotechnological potential of PGPRs isolated from maize in San Martín, Peru, revealed a remarkable diversity among the twelve strains analyzed. Rhizobacteria were isolated from the provinces of Lamas, El Dorado, Picota, and Bellavista, with a proportion of 58.3% Gram-positive and 41.7% Gram-negative bacteria, which were predominantly bacilli (83.3%). All strains demonstrated biological nitrogen fixation capabilities and variable production of IAA, with B3 and P4 strains showing the highest levels of production. The B5 and SJ1 strains exhibited the highest phosphate solubilization levels, while NSM3 and P4 produced the greatest amounts of siderophores. Treatments with B5 and the combination B3 + NSM3 resulted in greater root lengths, and all combinations exceeded 95% seed germination. Inoculated seedlings showed better development than that of the uninoculated control, with B3 + NSM3 excelling in the weight of fresh roots and the aerial part. The B5 and NSM3 strains have particularly promising prospects for the future development of bacterial inoculants due to their high similarity with *Sporosarcina luteola* Y1 AB473560 and *Peribacillus frigoritolerans* DSM 8801T, respectively. The use of native rhizobacteria could increase agricultural yields sustainably, reduce reliance on chemical fertilizers, and protect the environment. Moreover, their application could enhance food security by boosting the productivity of essential crops such as maize. Integrating these rhizobacteria into agricultural practices represents a viable ecological strategy for addressing current challenges and ensuring sustainable food production in the future.

## Figures and Tables

**Table 1:** Characterization of rhizobacteria strains isolated from maize plants in cultivations located in the provinces of Lamas, El Dorado, Picota, and Bellavista in the San Martin region.

Strains of Rhizobacteria	Microscopic Characteristics		Colony Characteristics				Origin
	Gram	Shape	Shape	Edge	Elevation	Consistency	
L2	-	Bacillus	Irregular	Undulated	Raised	Creamy	Lamas
L3-1	-	Bacillus	Circular	Entire	Raised	Creamy	Lamas
L4	-	Bacillus	Circular	Entire	Raised	Creamy	Lamas
L6	-	Bacillus	Circular	Entire	Raised	Creamy	Lamas
NSM3	+	Bacillus	Circular	Entire	Raised	Creamy	El Dorado
B3	+	Bacillus	Irregular	Entire	Raised	Creamy	Picota
B5	+	Bacillus	Circular	Entire	Raised	Creamy	Picota
P1	+	Cocci	Circular	Entire	Flat	Creamy	Picota
P3	+	Bacillus	Circular	Entire	Raised	Creamy	Picota
P4	-	Bacillus	Circular	Entire	Raised	Creamy	Picota
SJ1	-	Bacillus	Circular	Entire	Flat	Yellow	Bellavista
SJ2	-	Cocci	Circular	Entire	Flat	Creamy	Bellavista

**Table 2:** Plant growth-promoting characteristics of rhizobacteria strains isolated from maize plants cultivated in the provinces of Lamas, El Dorado, Picota, and Bellavista.

Strains of Rhizobacteria	BNF		Production of IAA (μg mL^−1^)	Solubilization of AlPO_4_ (μg mL^−1^)	Production of Siderophores (%)
	JMV	Burk			
L2	+	+	6.04 ± 1.03 fg	150.57 ± 1.48 g	5.95 ± 1.08 d
L3-1	+	+	1.15 ± 2.03 g	146.30 ± 2.96 gh	2.70 ± 1.87 d
L4	+	+	8.52 ± 1.98 fg	153.56 ± 0.00 g	8.47 ± 0.62 d
L6	+	+	4.57 ± 4.42 g	181.77 ± 7.80 e	3.06 ± 0.62 d
NSM3	+	+	26.94 ± 0.33 d	193.31 ± 3.39 d	89.19 ± 0.00 a
B3	+	+	44.54 ± 0.45 a	180.91 ± 4.12 e	32.61 ± 7.97 c
B5	+	+	35.65 ± 1.14 bc	233.91 ± 1.96 a	34.05 ± 2.86 c
P1	+	+	31.79 ± 4.51 cd	208.26 ± 1.96 c	10.99 ± 4.87 d
P3	+	+	36.21 ± 3.50 bc	165.96 ± 1.48 f	9.91 ± 1.25 d
P4	+	+	41.59 ± 3.39 ab	165.96 ± 0.74 f	57.12 ± 1.25 b
SJ1	+	+	19.10 ± 0.89 e	224.08 ± 2.22 b	10.63 ± 2.50 d
SJ2	+	+	12.60 ± 3.12 ef	192.88 ± 0.74 d	7.03 ± 1.08 d
Positive control	+	+	NE	141.60 ± 1.48 h	NE
CV (%)			11.88	1.68	13.45

BNF = Biological nitrogen fixation. JMV and Burk = Culture media. + = Growth in culture medium. Positive control = *Rhizobium tropici* CIAT 899. NE = Not evaluated. Tukey test (*p* < 0.05), means (n = 3) with different letters differ statistically from each other. CV: Coefficient of variation.

**Table 3:** Percentage of similarity of the 16S rRNA gene among rhizobacterial strains isolated from maize plants cultivated in the San Martin region.

Strains of Rhizobacteria	Place of Origin	Host	Most Related Species	Similarity (%)	Identified Strain/ Accession Number in NCBI GenBank
B5	Picota, Barranquita	*Zea mays*	*Peribacillus frigoritolerans* DSM 8801T	99.93	*Peribacillus* sp. B5/OP861655
NSM3	El Dorado, Nuevo San Martin	*Zea mays*	*Sporosarcina luteola* Y1 AB473560	99.44	*Sporosarcina* sp. NSM3/OP861656

**Table 4:** Locations of the sampling zones for soil and rhizospheric root samples from maize plants in the San Martin region.

Province	Sector	Coordinates		Altitude (masl)
		South Latitude	West Longitude	
Lamas	Huapo	06°22.869′ S	076°32.883′ W	536 m
	Alto Pucalpillo	06°25.204′ S	076°33.536′ W	680 m
	Cochapata	06°22.869′ S	076°32.052′ W	584 m
Picota	Barranquita	06°54.346′ S	076°18.226′ W	216 m
	Ponaza	06°54.126′ S	076°15.660′ W	229 m
	Ponaza 1	06°54.337′ S	076°15.407′ W	246 m
El Dorado	Nuevo San Martin	06°41.672′ S	076°37.250′ W	312 m
Bellavista	San Jose	07°02.077′ S	076°29.306′ W	233 m

## Data Availability

The molecular datasets generated during the study are available in NCBI GenBank under accession numbers OP861655 and OP861656. Other datasets used during the current study are available from the corresponding author upon reasonable request.

## Supplementary Materials

The following supporting information can be downloaded at: https://www.mdpi.com/article/10.3390/plants13152075/s1, Table S1: Effect of the inoculation with strains B3, B5, and NSM3, as well as their combinations (B3 + B5, B3 + NSM3, B5 + NSM3, and B3 + B5 + NSM3), under different nitrogen doses on the developmental parameters of maize seedlings (*Zea mays* L.) grown under gnotobiotic conditions, evaluated 15 days post-inoculation.
