# Supplementary material for: The Biotechnological Potential of Plant Growth-Promoting Rhizobacteria Isolated from Maize (Zea mays L.) Cultivations in the San Martin Region, Peru
Source: Plants (Basel). 2024 Jul 26;13(15):2075. doi: 10.3390/plants13152075 (PMC11313924; doi:10.3390/plants13152075)
Supplement: Supplementary file 1 [file plants-13-02075-s001.zip › plants-3123442-supplementary.pdf]

**Table S1**. Effect of the inoculation with strains B3, B5, and NSM3, as well as their combinations (B3 + B5, B3 + NSM3, B5 + NSM3, and B3 + B5 + NSM3), under different nitrogen doses on the developmental parameters of maize seedlings (*Zea mays* L.) grown under gnotobiotic conditions, evaluated 15 days post-inoculation.

| **Nitrogen doses (%)** | **B3** | **B5** | **NSM3** | **B3 + B5** | **B3 + NSM3** | **B5 + NSM3** | **B3+B5+ NSM3** | **Control** | **CV %** |
|---|---|---|---|---|---|---|---|---|---|
| | | | | **Root length (cm)** | | | | | |
| 0 | 18.65 ± 1.44 A$^{a}$ | 14.22 ± 1.14 A$^{de}$ | 14.95 ± 0.67 A$^{cde}$ | 17.65 ± 1.33 A$^{ab}$ | 13.82 ± 0.71 B$^{e}$ | 16.10 ± 2.02 Ab$^{cd}$ | 16.52 ± 1.58 A$^{bc}$ | 14.72 ± 2.16 A$^{cde}$ | 9.56 |
| 50 | 15.92 ± 1.31 B$^{ab}$ | 13.95 ± 2.51 A$^{c}$ | 16.70 ± 0.91 A$^{a}$ | 15.22 ± 1.22 A$^{abc}$ | 14.73 ± 0.36 AB$^{bc}$ | 15.65 ± 2.22 A$^{abc}$ | 16.08 ± 0.92 A$^{ab}$ | 14.53 ± 1.83 A$^{bc}$ | 9.63 |
| 75 | 15.42 ± 1.11 B$^{ab}$ | 14.38 ± 2.28 A$^{b}$ | 15.62 ± 1.45 A$^{ab}$ | 15.80 ± 1.62 A$^{ab}$ | 15.25 ± 1.53 AB$^{ab}$ | 15.50 ± 1.82 A$^{ab}$ | 16.78 ± 0.94 A$^{a}$ | 15.07 ± 1.74 A$^{b}$ | 10.94 |
| 100 | 14.85 ± 1.18 B$^{ab}$ | 14.22 ± 1.14 A$^{b}$ | 15.68 ± 2.18 A$^{ab}$ | 15.38 ± 2.12 A$^{ab}$ | 16.05 ± 1.71 A$^{ab}$ | 16.02 ± 1.33 A$^{ab}$ | 16.53 ± 1.91 A$^{a}$ | 14.63 ± 1.22 A$^{ab}$ | 9.37 |
| CV (%) | 8.29 | 12.65 | 8.42 | 10.43 | 8.65 | 12.35 | 9.22 | 13.57 | 10.64 |
| | | | | **Length of the aerial part (cm)** | | | | | |
| 0 | 73.38 ± 4.12 A$^{a}$ | 65.02 ± 5.20 A$^{c}$ | 71.03 ± 2.28 A$^{ab}$ | 68.92 ± 2.41 A$^{abc}$ | 58.35 ± 5.72 A$^{d}$ | 66.20 ± 4.07 B$^{bc}$ | 71.05 ± 2.76 A$^{ab}$ | 65.28 ± 5.46 A$^{c}$ | 6.43 |
| 50 | 70.32 ± 5.65 A$^{a}$ | 67.98 ± 2.11 A$^{ab}$ | 67.72 ± 5.89 A$^{ab}$ | 69.00 ± 2.33 A$^{a}$ | 71.12 ± 1.10 A$^{a}$ | 68.02 ± 3.23 AB$^{ab}$ | 71.30 ± 0.76 A$^{a}$ | 63.80 ± 2.21 A$^{b}$ | 5.17 |
| 75 | 68.97 ± 4.36 A$^{a}$ | 67.02 ± 5.15 A$^{a}$ | 68.38 ± 3.87 A$^{a}$ | 67.45 ± 8.39 A$^{a}$ | 60.08 ± 2.63 A$^{a}$ | 70.12 ± 2.14 AB$^{a}$ | 71.82 ± 3.66 A$^{a}$ | 62.38 ± 5.16 A$^{a}$ | 14.30 |
| 100 | 68.15 ± 1.72 A$^{ab}$ | 67.60 ± 0.94 A$^{bc}$ | 69.22 ± 2.23 A$^{ab}$ | 69.95 ± 7.01 A$^{ab}$ | 70.28 ± 1.44 A$^{ab}$ | 72.05 ± 1.60 A$^{a}$ | 69.17 ± 3.43 A$^{ab}$ | 63.85 ± 4.56 A$^{c}$ | 4.75 |
| CV (%) | 6.12 | 5.29 | 6.01 | 9.22 | 18.32 | 4.07 | 3.69 | 7.65 | 8.60 |
| | | | | **Fresh root weight (g)** | | | | | |
| 0 | 0.491 ± 0.18 A$^{ab}$ | 0.405 ± 0.08 C$^{b}$ | 0.668 ± 0.22 A$^{a}$ | 0.692 ± 0.21 A$^{a}$ | 0.638 ± 0.12 B$^{a}$ | 0.537 ± 0.09 A$^{ab}$ | 0.501 ± 0.17 A$^{ab}$ | 0.716 ± 0.25 A$^{a}$ | 30.60 |
| 50 | 0.552 ± 0.20 A$^{b}$ | 0.591 ± 0.11 AB$^{b}$ | 0.553 ± 0.17 A$^{b}$ | 0.452 ± 0.07 AB$^{b}$ | 0.805 ± 9.11 AB$^{a}$ | 0.561 ± 0.10 A$^{b}$ | 0.625 ± 0.18 A$^{ab}$ | 0.784 ± 0.18 A$^{a}$ | 24.21 |
| 75 | 0.426 ± 0.13 A$^{c}$ | 0.470 ± 0.15 BC$^{bc}$ | 0.483 ± 0.04 A$^{bc}$ | 0.428 ± 0.15 AB$^{c}$ | 0.824 ± 0.12 A$^{a}$ | 0.645 ± 0.13 A$^{ab}$ | 0.511 ± 0.22 A$^{bc}$ | 0.743 ± 0.22 A$^{a}$ | 29.20 |
| 100 | 0.604 ± 0.21 A$^{ab}$ | 0.667 ± 0.08 A$^{ab}$ | 0.598 ± 0.17 A$^{b}$ | 0.404 ± 0.14 B$^{c}$ | 0.783 ± 0.09 AB$^{a}$ | 0.587 ± 0.11 A$^{b}$ | 0.410 ± 0.17 A$^{c}$ | 0.707 ± 0.18 A$^{ab}$ | 23.81 |
| CV (%) | 32.48 | 20.37 | 32.72 | 33.08 | 14.56 | 18.12 | 30.73 | 30.92 | 28.15 |

| | | | | | | | | | |
|---|---|---|---|---|---|---|---|---|---|
| **Fresh weight of the aerial part (g)** | | | | | | | | | |
| 0 | 1.461 ± 0.29 A$^{a}$ | 1.467 ± 0.28 A$^{a}$ | 1.749 ± 0.78 A$^{a}$ | 1.578 ± 0.21 A$^{a}$ | 1.558 ± 0.32 B$^{a}$ | 1.773 ± 0.17 AB$^{a}$ | 1.401 ± 0.23 A$^{a}$ | 1.346 ± 0.38 A$^{a}$ | 24.44 |
| 50 | 1.666 ± 0.38 A$^{bc}$ | 1.769 ± 0.21 A$^{b}$ | 1.563 ± 0.37 A$^{bc}$ | 1.431 ± 0.18 A$^{c}$ | 2.339 ± 0.14 A$^{a}$ | 1.625 ± 0.10 B$^{bc}$ | 1.885 ± 0.25 A$^{b}$ | 1.420 ± 0.25 A$^{c}$ | 15.15 |
| 75 | 1.370 ± 0.26 A$^{cd}$ | 1.620 ± 0.35 A$^{bc}$ | 1.573 ± 0.39 A$^{bc}$ | 1.087 ± 0.62 A$^{d}$ | 2.376 ± 0.19 A$^{a}$ | 1.949 ± 0.20 AB$^{ab}$ | 1.622 ± 0.44 A$^{bc}$ | 1.613 ± 0.36A$^{bc}$ | 22.98 |
| 100 | 1.631 ± 0.44 A$^{abc}$ | 1.754 ± 0.24 A$^{ab}$ | 1.797 ± 0.32 A$^{ab}$ | 1.279 ± 0.21 A$^{c}$ | 2.029 ± 0.22 A$^{a}$ | 2.030 ± 0.29 A$^{a}$ | 1.474 ± 0.50 A$^{bc}$ | 1.564 ± 0.41 A$^{abc}$ | 20.99 |
| CV (%) | 20.29 | 14.62 | 24.52 | 24.65 | 10.94 | 11.23 | 20.36 | 23.98 | 22.07 |
| **Root dry weight (g)** | | | | | | | | | |
| 0 | 0.125 ± 0.03 A$^{a}$ | 0.098 ± 0.02 A$^{a}$ | 0.122 ± 0.02 A$^{a}$ | 0.120 ± 0.02 A$^{a}$ | 0.114 ± 0.05 A$^{a}$ | 0.103 ± 0.02 A$^{a}$ | 0.111 ± 0.03 A$^{a}$ | 0.109 ± 0.01 AB$^{a}$ | 22.77 |
| 50 | 0.115 ± 0.01 A$^{ab}$ | 0.125 ± 0.02 A$^{a}$ | 0.114 ± 0.02 A$^{ab}$ | 0.116 ± 0.02 A$^{ab}$ | 0.113 ± 0.01 A$^{ab}$ | 0.113 ± 0.01 A$^{ab}$ | 0.096 ± 0.01 A$^{b}$ | 0.098 ± 0.01 B$^{b}$ | 13.81 |
| 75 | 0.128 ± 0.02 A$^{ab}$ | 0.120 ± 0.02 A$^{ab}$ | 0.113 ± 0.03 A$^{b}$ | 0.138 ± 0.02 A$^{a}$ | 0.119 ± 0.01 A$^{ab}$ | 0.108 ± 0.01 A$^{b}$ | 0.115 ± 0.01 A$^{ab}$ | 0.105 ± 0.01 AB$^{b}$ | 15.04 |
| 100 | 0.153 ± 0.04 A$^{a}$ | 0.116 ± 0.02 A$^{bc}$ | 0.119 ± 0.01 A$^{b}$ | 0.115 ± 0.01 A$^{bc}$ | 0.094 ± 0.01 A$^{c}$ | 0.108 ± 0.01 A$^{bc}$ | 0.112 ± 0.03 A$^{bc}$ | 0.123 ± 0.02 A$^{b}$ | 15.73 |
| CV (%) | 19.13 | 18.26 | 16.09 | 16.75 | 24.46 | 12.61 | 17.24 | 13.51 | 18.38 |
| **Dry weight of the aerial part (g)** | | | | | | | | | |
| 0 | 0.156 ± 0.02 A$^{b}$ | 0.148 ± 0.02 A$^{bc}$ | 0.185 ± 0.02 A$^{a}$ | 0.144 ± 0.02 A$^{bc}$ | 0.114 ± 0.03 C$^{d}$ | 0.136 ± 0.02 A$^{bcd}$ | 0.155 ± 0.02 A$^{b}$ | 0.125 ± 0.02 A$^{cd}$ | 14.16 |
| 50 | 0.165 ± 0.02 A$^{abc}$ | 0.170 ± 0.01 A$^{ab}$ | 0.148 ± 0.02 A$^{c}$ | 0.154 ± 0.01 A$^{bc}$ | 0.180 ± 0.02 AB$^{a}$ | 0.147 ± 0.02 A$^{c}$ | 0.152 ± 0.02 A$^{bc}$ | 0.110 ± 0.01 A$^{d}$ | 10.32 |
| 75 | 0.159 ± 0.04 A$^{ab}$ | 0.160 ± 0.04 A$^{ab}$ | 0.152 ± 0.03 A$^{ab}$ | 0.160 ± 0.03 A$^{ab}$ | 0.188 ± 0.02 A$^{a}$ | 0.161 ± 0.02 A$^{ab}$ | 0.169 ± 0.02 A$^{ab}$ | 0.134 ± 0.02 A$^{b}$ | 17.01 |
| 100 | 0.156 ± 0.02 A$^{ab}$ | 0.158 ± 0.02 A$^{ab}$ | 0.166 ± 0.01 A$^{a}$ | 0.145 ± 0.01 A$^{ab}$ | 0.152 ± 0.02 B$^{ab}$ | 0.160 ± 0.01 A$^{ab}$ | 0.165 ± 0.03 A$^{a}$ | 0.133 ± 0.03 A$^{b}$ | 13.60 |
| CV (%) | 14.39 | 13.59 | 14.54 | 14.13 | 11.54 | 11.01 | 11.54 | 15.26 | 15.46 |
